# Beyond Self-Report: Emerging Methods for Capturing Individual Differences in Decision-Making Process

**DOI:** 10.3389/fpsyg.2016.00312

**Published:** 2016-03-03

**Authors:** Brenda L. Connors, Richard Rende, Timothy J. Colton

**Affiliations:** ^1^Chief of Naval Operations Strategic Studies GroupNewport, RI, USA; ^2^Social Behavioral Research ApplicationsPhoenix, AZ, USA; ^3^Department of Government, Harvard University, Cambridge, MAUSA

**Keywords:** decision-making style, decision-making scenarios, neuroscience, observational methods, movement

## Abstract

People vary in the way in which they approach decision-making, which impacts real-world behavior. There has been a surge of interest in moving beyond reliance on self-report measures to capture such individual differences. Particular emphasis has been placed on devising and applying a range of methodologies that include experimental, neuroscience, and observational paradigms. This paper provides a selective review of recent studies that illustrate the methods and yield of these approaches in terms of generating a deeper understanding of decision-making style and the notable differences that can be found across individuals.

## Introduction

That people differ in how they go about making decisions is not a new idea. What had been lacking in research, until recent years, was a dedicated effort to prioritize and focus intensively on capturing this fundamental variation in decision-making style (Mohammed and Schwall, 2009; Appelt et al., 2011). The issue is not just academic, as differential use of decision-making processes can have substantial implications for professional performance and career trajectories, health and well-being, and morbidity risk (e.g., Rosenbloom et al., 2012).

Connors et al. (2013) proposed that one way to advance our understanding of individual variation in decision-making was to supplement the reliance on self-report measures with concerted utilization of complementary approaches. The purpose of this paper is to highlight recent work that has gone beyond self-report to bring to light the profound variation that can be found in decision-making by using experimental, neuroscience, and observational methodologies. Our goal is not to provide an exhaustive review of the research literature, but rather to showcase selective studies that illuminate emerging themes that may serve as a catalyst for future research efforts. In addition, we illustrate the many ways decision-making process has been articulated within each discipline—there is as yet a universal language though many constructs share similarities—which may set a foundation for stimulating cross-disciplinary thinking.

## Why Move Beyond Self-Report?

There are certainly advantages to using a self-reported measure. It provides a quick and inexpensive way to gather information on decision-making style. Much of the earlier work on decision-making style focused on either evolution of specific questionnaires or application of existing self-report inventories that can yield information relevant to decision-making (see Appelt et al., 2011).

There has recently been a turn toward research that moves beyond self-report and focuses on alternative ways of capturing individual differences in decision-making style. Given the apparent advantages of self-report, why has this change occurred? One issue has to do with the predictive value of self-report. While self-perceptions can be quite illuminating, there have been suggestions that limitations be considered (e.g., Connors et al., 2013). For example, based on research with college students, Galotti et al. (2014) found that self-reports of decision-making style reflected how people viewed themselves as a decision maker, which was not highly correlated with objective recordings of their behavior. While self-perception matters, it is important to appreciate that it may not always be the best predictor of actual behavior.

Second, it has been noted (Appelt et al., 2011) that while prior research typically leaned on self-report measures to capture decision-making, only some—like the General Decision-Making Style or GDMS and the Rational–Experiential Inventory or REI—were developed with that specific purpose in mind. It has thus been proposed that other methodologies would be better suited to probe intensively into cognitive and motivational processes that underlie individual differences in decision-making (Connors et al., 2013).

We are seeing a concentration of a number of alternative and complementary research approaches being used that strive to gain more precise knowledge about decision-making process and how it varies substantially and systematically across individuals. Here we highlight three such approaches.

## Experimental Paradigms and Variation in Decision-Making Process

A direct way to study individual differences is to record how people differ when performing decision-making tasks, most typically in the laboratory setting under experimental conditions. Not many years back, it was suggested that this opportunity was highly under-utilized for two reasons (Mohammed and Schwall, 2009; see also Connors et al., 2013). First, many of the tasks were highly structured and offered participants very limited choices, which could mask differences between participants. Second, the statistical focus was geared toward how analyzing average scores for groups of subjects across tasks rather than systematic examination of how individuals varied within tasks. As a result, the methodologies truncated opportunities for individuals to vary, and statistical approaches that examine variation were not typically employed.

Over the last few years, we have seen application of methods that attend to these considerations by offering opportunities for expression of individual differences, and provide empirical demonstration of their prominence. One approach is to craft semi-structured experimental protocols that allow participants more freedom to direct their own decision-making process as observed in the laboratory. For example, Connors et al. (2013) developed four hypothetical decision-making scenarios (financial, health, voting, and strategy) in which participants were given options to seek out (one at a time) additional pieces of information that could be used to come to a final decision as required by the task. This method gave rise to substantial variation in metrics of decision-making process, including the number of pieces of information requested and the amount of total time devoted to arriving at a decision. Similarly, Del Missier et al. (2015) gave participants “poorly structured decision-making scenarios” which required them to generate their own options for choices in order to move toward a decision. Here three “option-generation problems”—solving a parking problem in a city, a fund raising issue in a non-profit organization, and a domestic energy saving situation—were presented, and the task was to generate as many options to consider as possible solutions within a fixed time period. The authors reported notable variation in option generation that was linked to a number of underlying cognitive processes. This study provides more evidence of the utility of intentionally designing decision-making scenarios as part of an effort to conduct an “individual-differences study” (Del Missier et al., 2015).

A primary issue raised by the use of hypothetical scenarios is the extent to which they are predictive of real-world decision-making (Parker et al., 2015). Parker and Fischhoff (2005) provided evidence that clinical and behavioral indicators of good decision-making by adolescents were predicted by commonly used decision tasks administered in the laboratory. More recently, Parker et al. (2015) reported that a hypothetical decision task—the adult decision-making competence (A-DMC) measure—was associated with a number of real-world decision outcomes, as recorded using the decision outcomes inventory (DOI).

Such evidence of predictive validity speaks to the utility of hypothetical decision-making scenarios, especially when incorporating methods for evaluating predictive power in real world settings. In addition, attention should be given to applying such approaches to a variety of populations of interest in order to provide opportunities to appreciate variability both within particular sub-populations (e.g., college students, physicians, investment bankers, military leaders) and across broader sampling of individuals from a variety of backgrounds (e.g., Parker et al., 2015).

## Behavioral Neuroscience and Individual Differences in Decision-Making

Another prominent theme that is populating the research literature is to use behavioral neuroscience methods to identify specific neurocognitive mechanisms and processes that underlie variation in decision-making. A number of studies published over the last few years have greatly advanced our understanding, and here we summarize a few to illustrate the type of progress to be made.

Laboratory protocols that offered broad choice in the decision-making process can reveal neural correlates of individual differences. For example, White et al. (2014) used a stop-signal task (which requires participants to inhibit, on demand, a quick response to a designated signal presentation) to activate neural systems that reflect individual differences in inhibitory control. They reported that this approach—particularly the “go” trials—provided insight into the decision components that are involved in inhibitory control, as well as differential brain activation (measured using fMRI) in the right parietal cortex that is associated with individual differences in motor execution time. Especially notable was their differentiation between “decision time” components (such as response caution or speed/accuracy tradeoff) and “non-decision time” elements (including encoding time and motor time). Such careful dissection of real-time processing brings important insight into all the elements that directly, and indirectly, impact decision-making, including some of the sources of differences across individuals.

Recent work by other investigators (Perri et al., 2014) took a similar approach using electroencephalographic (EEG) recordings in the go/no-go task, and demonstrated the individual differences in the fundamental balance between speed and accuracy during decision-making situations may be mediated in part by different (but interacting) neural systems. Taken together, these studies reflect current approaches that combine a laboratory task, neural recording, and an individual differences framework to gain insight into how people differ in fundamental neurocognitive processing that comprises aspects of decision-making.

This line of research also points in the direction of new insights being generated on the neural pathways that reveal multiple levels of complex processing which characterize how and why people differ. Doll et al. (2015) offer one illustration by focusing on what they describe as multiple decision systems, each of which may be under the control of a different brain region (e.g., the striatum versus the hippocampus). They showed that individual differences were elicited by employing different behavioral tasks that reflected these different computational strategies. The importance of this type of approach and similarly, guided research inquiries (see Connors et al., 2015) is to begin to specify stages and processes that comprise the entirety of the decision-making process, in the hopes of gaining insight into the various levels of analysis that can characterize the substantial observed variation in decision-making process and neural substrates.

In addition, a most exciting direction is to uncover different brain pathways that underlie variation in decision-making propensities using measures of functional connectivity (e.g., Vaidya and Gordon, 2013), which can be viewed as a way of understanding temporal and functional interrelationships within correlated neural networks. For example, van den Bos et al. (2014) focused on individual differences in “intertemporal decision-making”—making choices between immediate and delayed reward—and explored if functional (and structural) connectivity could reveal neural networks that may distinguish people who rely on patience versus impulsivity when faced with a task that pulls for delay of gratification. They reported evidence supporting this proposition, as they uncovered one anatomical/functional network that supported patience (functional connectivity between striatum and the lateral prefrontal cortex), and another associated with impulsivity (striatum and subcortical areas). As they suggest, such work highlights the possibility of gaining a deeper understanding of individual differences in aspects of decision-making that are mediated by distinctive anatomical and functional neural circuits (see also Rosenbloom et al., 2012).

Barnes et al. (2014) have offered a similar perspective that brings to the surface themes common to both experimental design and neural process. They adapted a widely used methodology—motion direction discrimination—to elicit individual differences in decision-making strategy that become apparent when subjects are given extensive time to make a decision (across two days). Spontaneous (or at rest) brain activity was predictive of decision-making strategies that varied across subjects, particularly the tendency to either accumulate evidence proactively to “expedite” the decision versus taking a “wait and see” approach to look for decreases in task difficulty over time to facilitate a decision. This work hints at the possibility of “hard-wired” variation in neural organization and function that may underlie differences in decision-making style.

## Observational Methods and Decision-Making Style

Yet another way to complement self-report is to utilize a range of methods to analyze decodable behavioral signals that are embedded in the stream of real-world functioning. Here we use the term “observational” to refer to examining recordable verbal and non-verbal indicators of decision-making propensities.

One such example is integrative complexity (IC), which is one measure of the construct of cognitive complexity that has been used with regularity for decades by political and social psychologists as an indicator of a number of attributes including decision-making style (Suedfeld and Tetlock, 2014). As discussed in Suedfeld and Tetlock (2014), IC has been used primarily as a method for analyzing running text, including oral recordings, and has applicability for capturing, at a particular moment in time, the degree or level of complexity an individual exhibits when approaching a specific issue or situation. It has been used regularly to examine the decision-making style and related constructs in a range of experienced leaders (Suedfeld and Tetlock, 2014).

Cognitive complexity is notable in part because it explicitly approaches decision-making style as an enduring personality trait. The emphasis is to move away from thinking about universal decision-making tendencies to argue that people have preferences for levels of complexity that represent a spectrum. Such preferences can be elicited and recorded via a variety of techniques, such as those used to capture IC. The dedication to methodological rigor in measuring constructs like IC (Suedfeld and Tetlock, 2014) provides a good illustration of the opportunities (and challenges) of observational work, and also offers evidence (accrued across decades) supporting the utility of exploring decision-making constructs as traits that vary across people.

Another observational approach has focused on human movement, specifically signature movement patterns which uncover how individuals prioritize motivational factors that guide their navigation through the decision-making process. We highlight here one method—movement pattern analysis (MPA)—which has been used for decades in the business world to guide, amongst many things, assessment of decision-making style of leaders and construction of management teams (Moore, 2005; Lamb, 2012). Application of MPA yielded robust indicators of decision-making style (and hence differences across individuals) that were highly predictive of variation in decision-making process as elicited by a number of hypothetical scenarios (Connors et al., 2013) with a high level of inter-rater reliability (Connors et al., 2014). MPA was also shown to be a sensitive indicator of individual differences in specific stages of the decision-making process which again predicted stylistic differences recorded while engaged in hypothetical scenarios (Connors et al., 2015). Such a focus on movement is consistent with the concept of embodied cognition (see Connors et al., 2013), and also corresponds to recent neurobiological work suggesting that the cerebellum has a complex role at the interface of movement, perception, and cognition (Koziol et al., 2014).

In terms of individual differences, this focus on staging offers perspective on capturing motivational proclivities when facing decisions. MPA measures how each individual cycles through decision-making processes along three major stages according to their motivational tendencies. The stages include Attending (focusing on information to become broadly and deeply informed), Intending (establishing a plan of action and prioritizing the goals within), and Committing (implementing the decision plan in a moment-to-moment pacing while envisioning how the stages of implementation unfold and impact the decision) – each of which is comprised of two action motivations associated with either Assertion (operational/tactical) and Perspective (strategic; Connors et al., 2013). MPA operationalizes each individual’s degree of motivation or propensity in each stage, which will differ across people even though all individuals will utilize each stage to some degree (Connors et al., 2015). People may begin and sequence through the decision making cycle very differently. For example, some people begin decision making by committing to the action and then afterwards return to Attend to details and Intend or justify their actions, whereas others have a great propensity to Attend (investigate and explore almost exclusively) and spend less in the Intending and Committing stages.

## Conclusion

This selective review has touched on a number of methods used to study individual differences in decision-making style and process that can complement more traditional self-report inventories. We conclude with a few broader considerations with respect to future directions for research.

First, there is a convergence across multiple fields of inquiry on the insight that variation in decision-making style matters greatly. In parallel, there is a surge of dedicated effort within each of these disciplines to develop sensitive methods that yield the greatest return in terms of appreciating both the scope and importance of variation in decision-making style. These methods tend to be more intensive measurement efforts than self-report. As noted earlier, our point is not to dismiss the self-report inventory; rather it is to appreciate the depth of study offered by other methodologies. To this point, future research could profitably begin to integrate self-report along with experimental, neuroscience, and observational paradigms to begin to gage the unique contributions of the different types of methods available. Indeed, tapping into each of these disciplines—using available approaches such as the ones highlighted in this paper—would represent a positive step toward a more multidisciplinary approach to variation in decision-making style and behavior.

Second, specific aspects of decision-making can certainly be the focus of such multidisciplinary science. One prime example concerns the study of decision-making under risk. Mishra (2014) has provided a compelling vision, integrating perspectives drawn from biology, psychology, and economics, focused on generating a more comprehensive multidisciplinary conceptual framework that can both integrate prior findings across fields and also promote future innovative research on decision-making under risk. Notably, Mishra (2014) also reviews what is described as robust empirical evidence for individual differences that must be accounted for by any theory of decision-making under risk.

We agree, and extend that suggestion to a multitude of dimensions of decision-making that can be the target of future research. More overt collaboration that crosses traditional boundaries of disciplines would stimulate integration of nomenclature and conceptions of the many cognitive processes that can be captured by a wide range of approaches, including laboratory, neuroscience, and observation methods. Taking such a path is especially likely to be fruitful given the common ground of appreciating both the scope and importance of individual differences captured by the wide scope of decision-making science that cuts across a number of disciplines.

## Author Contributions

BC and RR conceptualized paper, conducted review of literature, and wrote manuscript. TC contributed to writing and editing of manuscript.

## Conflict of Interest Statement

The authors declare that the research was conducted in the absence of any commercial or financial relationships that could be construed as a potential conflict of interest.
